# The epidemiology of homicide among older adults: retrospective analysis using data from the Victorian Homicide Register

**DOI:** 10.1007/s00414-023-03022-0

**Published:** 2023-05-29

**Authors:** Briohny Kennedy, Joseph Ibrahim, Sjaan Koppel, Lyndal Bugeja

**Affiliations:** 1 Department of Forensic Medicine, Monash University, 65 Kavanagh Street, Southbank, Melbourne, Victoria 3006 Australia; 2 Monash University Accident Research Centre, Monash University, Clayton, Australia

**Keywords:** Older adults, Homicide, Family violence, Social-ecological model, Violence prevention

## Abstract

Older adult homicide is unique and under-researched, requiring immediate attention due to the rapidly ageing population. The current study aims to contribute to the description of homicide at the individual, interpersonal, incident and community levels. This research comprised a whole of state jurisdiction population-based retrospective analysis of homicide deaths of older adults aged 65 years and older reported to the Coroner between 2001 and 2015. Descriptive statistical analyses were conducted to compare older adult homicides by sex and the deceased-offender relationship. There were 59 homicide incidents involving 23 female and 36 male deceased (median age=72 years) and 16 female and 41 male offenders (median age=41 years). Individual factors included the following: Deceased frequently had a recorded physical illness (66%), and over one-third were born overseas (37%) or had recent contact with general practitioners and human services (36%). Offenders frequently had a history of illicit drug or alcohol use (63%), diagnosed mental illness (63%) and historical exposure to violence (61%). Interpersonal factors included the following: The deceased-offender relationship tended to be intimate or familial (63%). Incidents factors included the following: incident predominantly occurred in the victim’s home (73%), involving the use of a sharp object (36%), bodily force (31%) or blunt force (20%). The older adult homicide is characterised by poor health in the victim, mental illness, substance abuse or a history of conflict in the either the victim or the offender, familial deceased-offender relationship and the home as the incident location. The results identify future prevention opportunities in clinical and human services settings.

**Supplementary Information:**

The online version contains supplementary material available at 10.1007/s00414-023-03022-0.

## Introduction

Preventing interpersonal violence is an international imperative [1]; the reduction of violence and associated death rates has received renewed priority within the United Nations Sustainable Development Goals [2]. Homicide represents the extreme of violence with substantive long-term and intergenerational impacts on individuals, communities and society [3, 4]. Due to physiological ageing and factors such as increased social isolation [5, 6], older adults may be more at risk and/or more vulnerable to assault or neglect than other age groups [7, 8]. It is expected that the global proportion of older adults aged 65 years and older will double from 2019 to 2050 [9]. This growth may well see older adult homicide become an increasing problem [10], and a failure to recognise older adults amongst the broader pool of homicide victims impacts the ability to provide adequate prevention strategies that might include policy and the allocation of human service resources [6, 10].

A recent meta-analysis reported a pooled older adult homicide rate of 2.02 per 100,000 [11], for the USA and other high-income Organisation for Economic Co-operation and Development (OECD) countries, and one that is lower than for younger adults aged 18–64 years (3.98 per 100,000). A study comparing mortality across the USA, Canada, Australia and New Zealand reported no decreasing trend in the older adult homicide rate between 2000 and 2012 [12]. Specifically in Australia, the National Homicide Monitoring Program reported that the 2018/2019 homicide rate had increased 16% on the previous year (2017/2018) and was the highest since the 2005–2006 reporting period [13].

Addington [10] recently identified the need for substantive descriptive research, in particular detailing offender characteristics, as well as in-depth analysis, that can support risk and protective factor identification for older adult homicide. There is also need for more detailed information about the fatal incident circumstances [6]. Additionally, further quantitative analysis related to potential risk factors is required [6], for example around themes such as mental health, drug and alcohol dependence, criminal history and carer dynamics as identified in recent older adult family homicide research [7, 14].

The stereotypical older adult homicide victim has been characterised as an older woman who is killed in their home by a stranger [6, 11]. In a recent meta-analysis, [11] the actual proportion estimate was lower for females than males (46.3 v 53.7%), and one-quarter (25.2%) were killed by family members, followed by strangers (24.2%), with an argument motive in 36.1% and a firearm mechanism in 24.5% of cases. When compared with younger adult homicides, older adult victims were more likely to be female (2.5-fold), and to have been killed by a stranger (1.8-fold), during a felony (2.8-fold), in their own home (3.9-fold) [11].

The familial deceased-offender relationship was explored in the UK, where older victims were also reported as primarily female, killed by a male spouse or a by a male adult child or grandchild [7], the latter being more likely than for younger adult victims [8]. Within the domestic homicide deceased-offender dyad, identified issues include financial stressors and a history of family violence [7, 14], as well as perpetrator mental health and drug and alcohol abuse [7].

An innovative process involves using the public health approach and a social-ecological framework modified to include an ‘incident’ level [11]. This allows multiple risk and protective factors associated with homicide to be interpreted with consideration of their interconnectedness [15].

Data generated for the criminal and coronial investigation of homicide is a valuable and rich information source [7, 16]. The objective of the current study was to examine the epidemiology of homicides among community-dwelling older adults and to describe typologies according to the deceased-offender relationship. This objective was addressed by answering two research questions:

1. What was the frequency of homicides among older adults in Victoria, Australia; and
2. What was the individual, interpersonal, incident, and community level factors identified among older adult homicides overall and when examined according to the deceased-offender relationship?

## Material and methods

### Study design

The design comprised a whole of state jurisdiction population-based retrospective analysis of homicides of older adults aged 65 years and older.

### Setting

The study setting was in the state of Victoria, Australia, during the period 2001 to 2015. In 2008, the mid-point of the study time period, 711,501 (13.5%) of the estimated Victorian population (*n*=5,256,375) were adults aged 65 years and older [17].

### Participants/case identification

Included were all deceased and offenders of older adult homicide where the deceased was aged 65 years and older, usually resided in the community, and where the coronial investigation had been completed at the time of data extraction (31 July 2017). Cases were identified using the Victorian Homicide Register (VHR) and study size was the whole population of deaths for the time period 1 January 2001 and 31 December 2015. (See Online Resource 1 for definitions.) One primary deceased and primary offender was identified for each unique homicide incident to avoid over-representation of individual homicide incidents in analysis.

### Variables

Variables were either extracted from the VHR or generated from existing variables (e.g. age in 10-year categories). These were organised within the levels of the modified social-ecological framework (Table 1).

**Table 1:** Variables included in the study according to modified social-ecological model

Social-ecological model level	Variables collected or derived from the Victorian Homicide Register
Individual (for both deceased and offender)	Demographic characteristics (i.e. age, sex); cultural and linguistic diversity factors (i.e. born overseas, language other than English, religion); recorded history of substance use or prior exposure to violence; diagnosed or suspected mental illness and treatment, physical illness diagnoses and treatment; and service contacts with government or non-government organisations (e.g. general practitioners/family physicians, emergency department, police, the courts or specialist domestic violence services).
Interpersonal	Deceased-offender relationship; age disparity; sex within dyad; motive; and family violence history.
Incident	Description of incident location (i.e. victim’s home); injury mechanism (i.e. firearm, sharp, blunt or bodily force); positive toxicology screen for alcohol or drugs (in the deceased), alcohol and/or illicit drugs detected in the offender; overkill (recorded evidence of excessive force, i.e. multiple stabbing, multiple violent methods or prolonged beating); and criminal justice outcomes.
Community	Socioeconomic Index for Areas (SEIFA) Index of Relative Socioeconomic Disadvantage (IRSD) scores and deciles, (www.abs.gov.au/websitedbs/censushome.nsf/home/seifa) Accessibility/Remoteness Index of Australia (ARIA) classifications (www.pocog.org.au/content.aspx?page=ariatool) for residence (deceased and offender) and incident location as proxy measures of disadvantage at the community level.

### Data source

Study data were obtained from the VHR, a prospective database (of deaths due to the assault or negligence) developed and maintained by the Coroners Court of Victoria (CCOV). The VHR contains coded and free-text information from the coronial brief, police summary of circumstances, autopsy and toxicology reports, sentencing remarks (where relevant), the coroners’ finding and where the homicide occurred between persons in an intimate or familial relationship, the Victorian Systemic Review of Family Violence Deaths report. This volume and detail of data make the VHR a useful information source for homicide research.

### Bias

Data for all VHR variables were entered by trained personnel working in the Coroners Prevention Unit, CCOV, in accordance with an established data dictionary, which was then reviewed by a second trained staff member for internal consistency. (Author BK was trained in coding into the VHR and coded 25 homicide incidents.)

The dataset is robust in its inclusivity and does not rely on voluntary reporting of homicides as reported by other research internationally [5].

### Statistical analysis

All descriptive outputs and analyses were performed using IBM SPSS Statistics for Windows, version 28.0 (IBM Corp., Armonk, NY, USA). Pearson chi-squared tests were used to compare deceased and offender characteristics by sex and deceased-offender relationship type. *T*-tests were used to compare continuous variables (i.e. age).

## Results

**Fig. 1:**
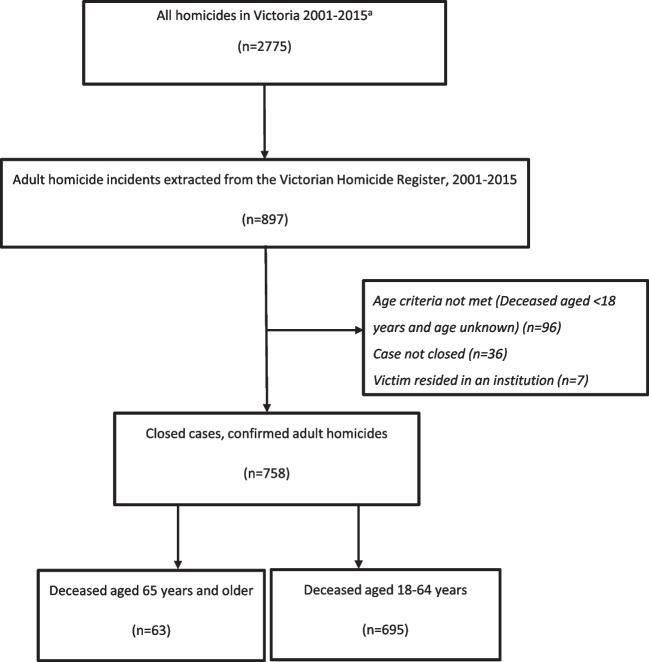
Case identification. a Source: Victoria Police Crime Statistics

During the period 2001–2015, there were 63 homicide deaths among adults aged 65 years and older (Fig. 1). Of these, there were 59 unique older adult homicide incidents: with 59 primary deceased victims (36 male, 23 female, Table 2) and 57 primary offenders (41 male, 16 female, Table 3). Homicide victims were more frequently younger than 75 years (*n*=37, 63%; median=72, IQR=11), while offenders were predominantly aged between 25 and 55 years (*n*=36, 63%; median=41, IQR=22).

**Table 2:** Overview of homicides among older adults (deceased), Victoria 2001–2015

Variable		Deceased (*n*=59)	%	Female (*n*=23)	%	Male (*n*=36)	%
Individual level							
Accommodation type	Private residence—owned/private rental	29	49.2	15	65.2	14	38.9
	Private residence—public rental	6	10.2	<5	-	5	13.9
Age group	65–74	37	62.7	13	56.5	24	66.7
	75–84	18	30.5	9	39.1	9	25
	85–94	<5	-	<5	-	<5	-
Any physical illness	Yes	39	66.1	14	60.9	25	69.4
Country of birth	Australia	15	25.4	<5	-	12	33.3
	Outside Australia	22	37.3	10	43.5	12	33.3
Diagnosed mental illness	Yes	8	13.6	<5	-	<5	-
Employment status	Employed	6	10.2	<5	-	5	13.9
	Retired/pensioner	44	74.6	18	78.3	26	72.2
Ethnic or cultural affiliation	Known	11	18.6	5	21.7	6	16.7
History of substance use	Yes	8	13.6	0	0	8	22.2
History violence exposure	Yes	15	25.4	6	26.1	9	25
Main language	English	23	39	5	21.7	18	50
	LOTE - known	9	15.3	<5	-	5	13.9
Mental illness, suspected	Yes	12	20.3	<5	-	8	22.2
Physical disability	Yes	4	6.8	<5	-	<5	-
Physical injury	Yes	11	18.6	<5	-	8	22.2
Psychiatric treatment (non-proximal)	Yes	6	10.2	5	21.7	<5	-
Psychiatric treatment (proximal)	Yes	11	18.6	7	30.4	<5	-
Service contact (any)	Yes	29	49.2	10	43.5	19	52.8
Service contact (proximal)	Yes	21	35.6	8	34.8	13	36.1
Suicide attempt	Yes	-	-	-	-	-	-
Suicide ideation	Yes	-	-	-	-	-	-
Undergoing treatment for physical illness	Yes	16	27.1	9	39.1	7	19.4
Interpersonal level							
Deceased-offender relationship	Friend/acquaintance	11	18.6	<5	-	9	25
	Intimate partner	10	16.9	<5	-	8	22
	Other familial	27	45.8	14	60.9	13	36.1
	Stranger	8	13.6	<5	-	5	13.9
	Unknown	<5	-	<5	-	<5	-
Family homicide	Yes	38	64.4	17	73.9	21	58.3
FV homicide	Yes	16	27.1	7	30.4	9	25
Motive argument	Yes	15	25.4	<5	-	11	30.6
Incident level							
Incident location	Deceased’s home	32	54.2	9	39.1	23	63.9
	Offender’s home	<5	-	<5	-	0	0
	Public place	10	16.9	<5	-	6	16.7
	Shared residence	10	16.9	5	21.7	5	13.9
Mechanism	Blunt object	12	20.3	5	21.7	7	19.4
	Bodily force	18	30.5	8	34.8	10	27.8
	Sharp object	21	35.6	7	30.4	14	38.9
Overkill (excessive force)	Yes	6	10.2	<5	-	<5	-
Presence of alcohol or drugs (deceased)	Yes	29	49.2	13	56.5	16	44.4
Community level							
Residence ARIA	Inner regional - outer regional	8	13.6	<5	-	6	16.7
	Major city - inner regional	50	84.7	20	87	30	83.3
Residence IRSD	1	6	10.3	<5	-	<5	-
	2	10	17.2	<5	-	8	22.2
	3	9	15.5	5	22.7	<5	-
	4	14	24.2	<5	-	10	27.8
	5	19	32.8	8	36.4	11	30.6

**Table 3:** Older adult homicide offenders, Victoria, 2001–2015

Variable	Categories	Offender (*n*=57)	%	Female (*n*=16)	%	Male (*n*=41)	%
**Individual level**							
Accommodation type	Private residence—owned/private rental	27	47.4	6	37.5	21	51.2
Age group	≤24	27	47.3	<5	-	6	14.6
	25–44	22	38.6	7	43.8	15	36.6
	45–64	17	29.9	<5	-	13	31.7
	65–84	6	10.6	<5	-	5	12.2
Any physical illness	Yes	12	21.1	5	31.3	7	17.1
Country of birth	Australia	24	42.1	6	37.5	18	43.9
	Outside Australia	14	24.6	<5	-	11	26.8
Diagnosed mental illness	Yes	36	63.2	12	75	24	58.5
Employment status	Employed	19	33.3	<5	-	16	39
	Unable to work	6	10.5	<5	-	<5	-
	Unemployed	15	26.3	<5	-	12	29.3
Ethnic or cultural affiliation	Yes	16	28.1	<5	-	13	31.7
History of substance use	Yes	36	63.2	11	68.8	25	61
History violence exposure	Yes	35	61.4	11	68.8	24	58.5
LGBTI identity	Yes	<5	-	<5	<5	<5	-
Main language	English	26	45.6	5	31.3	21	51.2
	LOTE - known	6	10.5	<5	-	5	12.2
Physical injury	Yes	6	10.5	<5	-	<5	-
Previous suicide attempt	Yes	11	19.3	5	31.3	6	14.6
Prior offending	Yes	27	47.4	7	43.8	20	48.8
Psychiatric treatment (non-proximal)	Yes	31	54.4	10	62.5	21	51.2
Recent psychiatric treatment	Yes	24	42.1	10	62.5	14	34.1
Service contact (any)	Yes	43	75.4	13	81.3	30	73.2
Service contact proximal	Yes	26	45.6	8	50	18	43.9
Suicidal ideation	Yes	12	21.1	6	37.5	6	14.6
Suspected mental illness	Yes	22	38.6	5	31.3	17	41.5
Treatment for physical illness	Yes	7	12.3	<5	-	<5	-
**Incident level**							
Offender charged	Yes	49	86	14	87.5	35	85.4
Offender sentenced	Yes	37	64.9	11	68.8	26	63.4
Presence of alcohol and/ or illicit drugs	Yes	24	42.1	8	50	16	39
**Community level**							
Residence ARIA	Inner regional - outer regional	5	8.8	<5	-	<5	-
	Major city - inner regional	47	82.5	12	75	35	85.4
Residence IRSD	1	6	11.5	<5	-	5	13.5
	2	9	17.3	<5	-	5	13.5
	3	11	21.1	<5	-	8	21.6
	4	9	17.3	<5	-	6	16.2
	5	17	32.7	<5	-	13	35.1

### Individual-level factors

#### Deceased

Among the 59 primary deceased homicide victims, six (10%) were employed at the time of their death (Table 2). Over one-third (*n*=22, 37%) were born overseas, of which nine (41%) had their main language recorded as not English. Two-thirds (*n*=39, 66%) had at least one diagnosed physical illness, of which 36% (*n*=14) were currently receiving treatment.

Almost 14% of deceased had a diagnosed mental illness recorded (*n*=8), and in a further 15% (*n*=9) a mental illness was suspected. There was a recorded history of alcohol and/or other illicit substance use for 14% (*n*=8). Eleven (19%) had been receiving psychiatric care proximal (within 6 weeks prior) to the homicide. Around one-quarter had experienced historical exposure to violence (*n*=15, 25%).

Almost half of the deceased (*n*=29) had previously been in contact with government or non-government human services, for most of which that contact was proximal (within 6 weeks prior) to the homicide (*n*=21, 36%). This contact was mainly with a general practitioner (GP)/family physician (*n*=10, 17%), social security (*n*=6, 10%) or other non-government agency (*n*= 5, 9%).

#### Offenders

Among the 57 primary offenders of older adult homicide, 19 (33%) were employed at the time of the homicide (Table 3). Of the 25% (*n*=14) of offenders that were born overseas, 43% (*n*=6) had their main language recorded as not English. Only 21% (*n*=12) had at least one diagnosed physical illness, of which 58% (*n*=7) had been receiving treatment.

Eighty-one per cent (*n*=46) of older adult homicide offenders had mental illnesses recorded as diagnosed (*n*=24), suspected (*n*=10) or a combination of diagnosed and suspected (*n*=12). Of the 36 with diagnosed mental illness, singular and multiple diagnoses were recorded, including substance use disorder (19, 53%), mood (affective) disorders (including bipolar affective disorder and depression) (*n*=17, 47%), schizophrenia, schizotypal and delusional disorders (*n*=10, 28%) and neurotic, somatoform and stress-related disorders (for example anxiety, PTSD and acute crisis) (*n*=9, 25%).

The majority had received psychiatric treatment at some time (*n*=28, 78%), with most receiving care proximal to the incident (*n*=21, 58%), most commonly by way of voluntary community treatment (*n*=17, 47%). Over half had a history of substance use (*n*=36, 63%) or history of exposure to violence (*n*=35, 61%).

Most offenders had previously been in contact with services (*n*=43, 75%), though less than one-half (*n*=26, 46%) had been proximal to the incident. Proximal service contacts included social services (*n*=14, 25%), police (*n*= 9, 16%), and family physicians/GPs, the law courts and drug and alcohol services (all 5, 9%).

Though differences were not statistically significant, female offenders had a higher incidence than males of historical exposure to violence (females *n*=11, 69%; males *n*=24, 59%), suicide ideation (females *n*=6, 38%; males *n*=6, 15%), suicide attempt (females *n*=5, 31%; males *n*=6, 15%), mental illness diagnoses (females *n*=12, 75%; males *n*=24, 59%), and proximal psychiatric treatment (females *n*=10, 63%; males *n*=14, 34%).

### Interpersonal-level factors

Homicide deceased were primarily more than 25 years older than their offender (*n*=37, 62%), with the difference ranging between 25 and 64 years (median=34, IQR=22) (Online Resource 2). The deceased-offender relationship was largely intimate or familial (*n*=37, 63%), followed by friends and acquaintances (*n*=11, 19%) and strangers (*n*=8, 14%). Argument motive accounted for 15 (25%) of older adult homicides (Table 2). Motive was described as mental impairments such as psychosis, delusional, and drug and alcohol intoxication occurring in 13 incidents (22%).

**Fig. 2:**
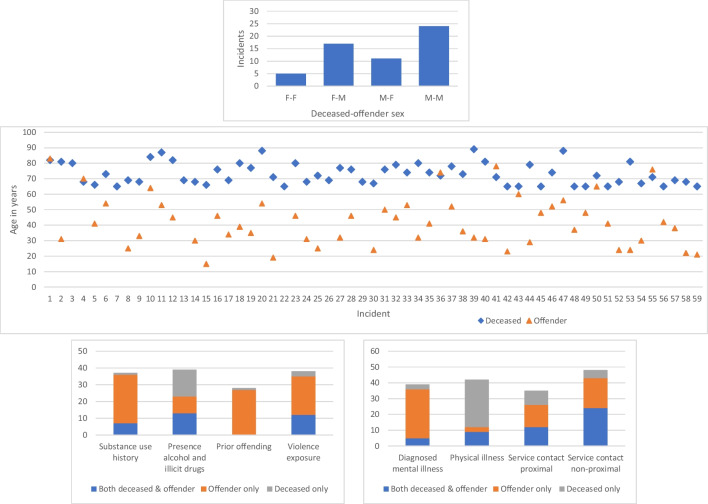
Dyadic (interpersonal) comparison of primary deceased and offender. The top graph depicts the number of incidents by sex within the deceased-offender relationship; the middle graph depicts the age of the deceased and offender by individual incident; and the bottom two graphs depict the presence of selected variables in either the deceased, the offender or both the deceased and offender, specifically: substance use history, presence of alcohol and drugs, prior offending, historical exposure to violence, diagnosed mental illness, physical illness, and proximal and non-proximal service contacts.

Age difference was common at the case by case level, and the more common interactions were a male deceased and a male offender (*n*=24, 41%) and a female deceased and a male offender (*n*=17, 29%) (Fig. 2). The comparison also highlights greater substance use history, prior offending, diagnosed mental illness, and historical exposure to violence for the offender and physical illness and proximal service contacts for the deceased (Fig. 2).

### Incident-level factors

The incident location was predominantly the deceased’s home (*n*=43, 73%). Primary injury mechanisms were sharp object (*n*=21, 36%), bodily force (*n*=18, 31%) and blunt object (*n*=12, 20%). There was a positive toxicology screen for 29 (49%) of the deceased and alcohol and/or illicit drugs were detected 24 (42%) of offenders at the time of the incident (Tables 2 and 3; Fig. 2). An offender was identified in almost all incidents (*n*=58, 98%), charged in 86% (*n*=49) and sentenced in 65% (*n*=37) of the homicides. In the 12 cases where the offender was not sentenced, the outcome was primarily not guilty by reason of mental impairment (*n*=9), followed by acquitted or not fit to stand trial (*n*<5).

### Community-level factors

Deceased victims commonly resided in a major city or a major city-inner regional area (*n*=50, 85% and *n*=47, 83%; Tables 2 and 3). The majority of victims and offenders were from the higher-level deciles (6^th^ to 10^th^) for IRSD, indicating that they resided in communities with lower levels of socioeconomic disadvantage (deceased *n*=36, 62%; offenders *n*=31, 60%).

### Older adult homicides by deceased-offender relationship

#### Homicide among persons in intimate or familial relationships

Among the 37 homicides that occurred among intimate partners (*n*=10) or family members (*n*=27), 48% (*n*=13) involved both a male deceased and offender (Online Resource 2). The home location was more frequent (*n*=31/37) and significantly different from the acquaintance (*n*=7/11) and stranger (*n*≤5/8) perpetrated homicides (Yates Continuity Correction 6.118 (1), *p*=.013, Phi .361). Compared with other relationship types, intimate or familial homicides involved a higher frequency of argument motive, and blunt object or bodily force mechanisms. A history of family violence was recorded for 15 (41%) incidents; eight deceased (22%) had been victims and seven (19%) were perpetrators of family violence.

Fourteen (38%) of the 37 intimate or familial homicides comprised a deceased parent killed by their adult child. The deceased were equally male or female (*n*=7), more offenders were male (*n*=10) and bodily force was the most common mechanism of injury (*n*=6). Within the parent-child relationship, 57% (*n*=8) of deceased had either perpetrated (*n*≤5) or been victims (*n*≤5) of violence.

Ten (27%) of the intimate or familial homicides occurred between intimate partners. More of the deceased were male (*n*=8), while offenders were equally divided between male and female (*n*=5). All incidents occurred at the deceased’s home or a residence shared with the offender. The most common mechanism of injury was a sharp object (*n*=5, 50%).

Homicides among other family members (*n*=13, 35%) included grandparents, aunts and uncles, in-laws and cousins, and more of the deceased were female (*n*=7, 54%). Eight (61%) were multiple fatality events (Online Resource 2).

#### Homicide among friends and acquaintances

Eleven (19%) of the 59 deceased were killed by friends and acquaintances. Over 80% of deceased were male (*n*=9, 82%), aged between 65 and 74 years (*n*=8, 73%), and were killed by other males (*n*=10, 91%). Over one-half of the deceased had been in contact with services at some time not proximal to the fatal incident (*n*=6, 55%) (Online Resource 2). These differed to intimate or familial and stranger relationship types in that the homicide occurred at the deceased’s home less often (*n*=7, 64%), was more often a single-fatality incident (*n*=10, 91%), involving a sharp object (*n*=6, 55%) and with offenders with an historical exposure to violence (*n*=9, 82%) and prior offending (*n*=7, 64%).

#### Homicide perpetrated by strangers

Eight (14%) of the 59 deceased were killed by strangers. The deceased were typically male (*n*=5, 63%) and aged between 65 and 74 years (*n*=5, 63%) (Online Resource 2). All stranger offenders had a substance use history, which was a significant difference to other relationship types (*n*=8, 100%; Yates Continuity Correction 3.743 (1), *p*=.021, Phi .309), and the incident location was significantly less frequently at home (Yates continuity correction 3.397(1), *p*=.037, Phi −.295). Offenders had more history of offending (*n*=6, 75%), diagnosed mental illness (*n*=7, 88%) and psychiatric treatment history (*n*=7, 88%) than for the other relationship types (Online Resource 2).

## Discussion

### Summary of key findings

The most common older adult homicide victim for this study was male (61%) killed in his own home (72%), by an intimate or family member (62%), with a sharp object (36%) or bodily force (31%). Many victims in this study had a high level of existing illness, and had been in contact with their family physician or human services prior to the incident. Most also knew their offender, of which diagnosed mental illness and prior exposure to violence and a history of substance use were recorded.

### Interpretation

#### Individual level

Two-thirds (66%) of the deceased victims had a diagnosed physical illness. A large proportion of the deceased (90%) and offenders (67%) were not in employment, mostly retired which may implicate financial stress, a key theme identified in adult family homicide research [7, 14]. Over one-third (36%) had been in recent contact with services, such as their family physician or social services, which offers a potential intervention setting.

The offender was primarily male; a high proportion had been receiving care for mental illness and almost half had recently been in contact with human services, police or other services. Compared to deceased older adults, their offenders had a higher frequency of reported prior offending, history of substance use and historical violence exposure. Offender diagnosed mental illness (63%) was substantially greater than that recorded for the deceased (14%). This is similar to recent findings on adult family homicide [14] and in line with research identifying greater mental illness in older adult homicide offenders than for homicides against other age groups [18].

#### Interpersonal level

Almost two-thirds of older adults were killed by someone substantially younger; however, older adults were also killed by their intimate partner. Argument motive was recorded for 25% of the homicides which was less than that reported in a recent meta-analysis [11]. Reasons might include differences in how motive is defined and recorded, but might also reflect unspecified structural differences between jurisdictions.

#### Incident level

Almost half of offenders had alcohol or illicit drugs detected post-homicide. The motive was identified as due to intoxication or due to a delusional or psychotic state in 22% of incidents. There were possibly warning signs and earlier opportunities to intervene or prevent these mental health crises, as previously identified in a thematic analysis of adult family homicide in the UK [14]. This level of information, however, was not available for the current study.

#### Community level

The majority of incidents occurred in major cities or inner regional areas. Both deceased and offenders also resided in areas classified as less socioeconomically deprived; a further detailed analysis would be required to determine the association of these potential community-level stressors with older adult homicide.

### Homicide type by deceased-offender relationship

Not surprisingly, intimate or familial older adult homicides most frequently occurred in the home, which supports findings from recent domestic homicide research [7, 8, 14]. This also supports lifestyle-routine activities theory research explaining that the older adult is more likely to be at home [11].

Contrasting to other intimate partner homicide research describing primarily male offenders [7, 13], we found older adult intimate partner homicide offenders to be equally male or female. More than half (8/13) of the non-intimate family homicides were multiple fatality events. Multiple fatality homicides sometimes involve the older parent as a corollary victim of intimate partner violence or homicide [19]. However, in our study, the majority of multiple-fatality events (*n*=7) were characterised by either multiple older victims, for example the killing of both parents, or a homicide followed the subsequent suicide of the offender (murder-suicide), usually by an adult child.

### Generalisability

While these results contribute to the empirical literature, findings should be generalised with caution outside of Australia due to cross-jurisdictional variation in violent crime legislation and enforcement, community attitudes and structural influences [11].

### Strengths and limitations

This study is the first to publish data from the VHR and, to our knowledge, present older adult homicide data according to a modified social-ecological model. Strengths of this novel study are that a large number of known homicide risk factors are explored using a quality data source, particularly for the offender and their relationship to the deceased. It includes all eligible homicides over a 15-year period, which is particularly rare for the richness of data.

The case closure process can introduce bias to the description of older adult homicide through missing cases in the dataset [16]. Additionally, despite the rigour employed with data entry, there is still the potential for human error, i.e. gaps in the investigation process or incorrect assumptions by the primary data collectors.

#### Diversity

As much information as possible was recorded in the database to identify important diverse subgroups. This included factors such as whether deceased or offenders belonged to specific ethnic groups, primarily spoke a language other than English, the nationality of individuals and their parents, physical ability, gender and sexual identity, religious affiliation and cultural groups. A caveat is that data collection was likely to be inconsistent for many of these variables, as source data were originally collected for the purpose of criminal investigation and homicide monitoring.

Older adult homicide is gendered in that key factors can differ between male and females, in particular the deceased victims and that there tends to be more offenders that are male. For transparency, we have presented male and female data both individually and together for deceased and offenders in Tables 2 and 3, and tested for statistical differences for these factors by sex.

There is always a probability that some homicide data may be incomplete, and it is possible that missing cases may be the result of persons not registered as citizens or having a fixed address (i.e. homeless) at the time of the incident [20].

### Implications

Strategies to aid the prevention of older adult homicide listed in the extant literature include screening caregivers [21], family interventions (e.g. caregiver support) [22, 23], security (e.g. neighbourhood watch) [5, 22, 24], awareness-raising and education [5, 18, 24], shelters [22] and improving medical response for assaulted people [5].

Violence prevention efforts in Australia, while addressing these aspects, have largely focussed on women and their children [25]. While there are also efforts to ensure older adults are included (for example through specialist elder abuse services), large-scale attitudinal campaigns have not directly addressed detrimental attitudes towards older adults (ageism) or issues impacting the older adult’s ability to access support.

## Conclusion

An older adult is potentially more vulnerable to homicide when living with an illness or disability, or residing in an intergenerational household with the presence of mental health diagnosis, substance abuse or a history of conflict in either the patient or their familial or close contacts. Recent contact with physicians and human services represents a potential future intervention opportunity. More studies describing the homicide incident at multiple levels are required to further validate these findings.

## Supplementary information

ESM 1

ESM 2

## Data Availability

Due to the sensitive nature of the research and subsequent ethical and legal limitations, the raw data from this study cannot be shared publicly.
