# Supplementary material for: The epidemiology of homicide among older adults: retrospective analysis using data from the Victorian Homicide Register
Source: Int J Legal Med. 2023 May 29;137(5):1583–93. doi: 10.1007/s00414-023-03022-0 (PMC10421821; doi:10.1007/s00414-023-03022-0)
Supplement: Supplementary file 1 — ESM 1 [file 414_2023_3022_MOESM1_ESM.docx]

**The epidemiology of homicide among older adults: Retrospective analysis using data from the Victorian Homicide Register**

**International Journal of Legal Medicine**

**Authors:** Briohny Kennedy^1^, Joseph Ibrahim1, Sjaan Koppel, PhD^2^, and Lyndal Bugeja.^1^

**Author affiliations:**

1 - Monash University Department of Forensic Medicine

2 - Monash University Accident Research Centre, Monash University

**Corresponding author:**

Ms Briohny Kennedy, 65 Kavanagh Street, Southbank, Victoria, 3006, Australia, Phone: +61 3 9684 4444, Email: [briohny.kennedy@monash.edu](mailto:briohny.kennedy@monash.edu)

**Online Resource S1** Definitions used in this study

A community dwelling adult was defined as living either in a private or publicly owned dwelling, or homeless at the time of the incident, and not in an institution (for example a prison, hospital, aged care home or other institutional/residential care).

An older adult was defined as a person aged 65 years and older in accordance with the Australian Government (Australian Institute of Health and Welfare, 2021) and more broadly in Australia and other OECD countries, for example the UK (National Health Service England, 2021) and the US (U.S. Department of Health and Human Services, 2021).

Homicide is defined in ICD-10 Version15 as ‘injuries inflicted by another person with intent to injure or kill, by any means’ that includes neglect and abandonment ([www.icd.who.int/browse10/2015/en#/X85](http://www.icd.who.int/browse10/2015/en#/X85)). For this study, we adopted the definition used by the CCOV for the VHR that includes the ‘unlawful killing of a person, where the death occurred or was suspected to have occurred due to external [non-natural] causes attributable to a person through assaultive force’. This definition includes: murder, manslaughter, murder-suicides, infanticides and homicides as classified by the Victorian Police regardless of offender apprehension or conviction, and includes those where the offender was excused from criminal liability due to mental impairment. This definition excludes: driving-related fatalities not proximal to a crime, industrial accidents, unless manslaughter charges are laid, lawful homicide (i.e. by police in the course of duty), and missing persons unless a murder or manslaughter charge was laid.

**References**

Australian Institute of Health and Welfare. (2021). *Older Australians* (Older people, Issue. A. Government. <https://www.aihw.gov.au/reports/older-people/older-australians>

National Health Service England. (2021). *Improving care for older people*. National Health Service England. Retrieved cited 26 January 2022 from <https://www.england.nhs.uk/ourwork/clinical-policy/older-people/improving-care-for-older-people/>

U.S. Department of Health and Human Services. (2021). *Aging*. U.S. Department of Health and Human Services. Retrieved cited 23 February 2022 from <https://www.hhs.gov/aging/index.html>
