# Supplementary material for: The epidemiology of homicide among older adults: retrospective analysis using data from the Victorian Homicide Register
Source: Int J Legal Med. 2023 May 29;137(5):1583–93. doi: 10.1007/s00414-023-03022-0 (PMC10421821; doi:10.1007/s00414-023-03022-0)
Supplement: Supplementary file 2 — ESM 2 [file 414_2023_3022_MOESM2_ESM.docx]

**The epidemiology of homicide among older adults: Retrospective analysis using data from the Victorian Homicide Register**

**International Journal of Legal Medicine**

**Authors:** Briohny Kennedy^1^, Joseph Ibrahim1, Sjaan Koppel, PhD^2^, and Lyndal Bugeja.^1^

**Author affiliations:**

1 - Monash University Department of Forensic Medicine

2 - Monash University Accident Research Centre, Monash University

**Corresponding author:**

Ms Briohny Kennedy, 65 Kavanagh Street, Southbank, Victoria, 3006, Australia, Phone: +61 3 9684 4444, Email: [briohny.kennedy@monash.edu](mailto:briohny.kennedy@monash.edu)

**Online Resource 2** Older adult homicide characteristics by deceased-offender relationship type

| **Variable** | **Categories** | **Total deceased (n=59)** | **(%)** | **Acquaintance (n=11)** | **Intimate or familial (n=37)** | **Stranger (n=8)** |
| --- | --- | --- | --- | --- | --- | --- |
| **Individual level** |  |  |  |  |  |  |
| Age group | 65-74 | 37 | 62.7 | 8 | 21 | 5 |
|  | 75-84 | 18 | 30.5 | <5 | 13 | <5 |
|  | 85-94 | <5 | - | - | <5 | <5 |
| History violence exposure | Yes | 15 | 25.4 | <5 | 9 | <5 |
| History of substance use | Yes | 8 | 13.6 | <5 | 5 | <5 |
| Psychiatric treatment (proximal) | Yes | 11 | 18.6 | <5 | 5 | <5 |
| Service contact (any) | Yes | 29 | 49.2 | 6 | 17 | <5 |
| Service contact (proximal) | Yes | 21 | 35.6 | 5 | 10 | <5 |
| Sex | Female | 23 | 39 | <5 | 16 | <5 |
|  | Male | 36 | 61 | 9 | 21 | 5 |
| **Interpersonal level** |  |  |  |  |  |  |
| Deceased ≥ 25 years older | Yes | 37 | 62.7 | 5 | 25 | 7 |
| Age disparity (in years) | D older 5-24 | 10 | 17 | <5 | 5 | <5 |
|  | D older 25-44 | 27 | 45.7 | <5 | 19 | <5 |
|  | D older 45-64 | 10 | 17 | <5 | 6 | <5 |
| History of FV in dyad^a^ | Yes | 16 | 27.1 | - | 15^b^ | - |
| FV homicide | Yes | 16 | 27.1 | - | 15^b^ | - |
| FV perpetrator^a^ | Yes | 8 | 13.6 | - | 7 | - |
| FV victim^a^ | Yes | 9 | 15.3 | - | 8 | - |
| Motive argument | Yes | 15 | 25.4 | <5 | 13 | - |
| Victim emotional abuse^a^ | Yes | 7 | 11.9 | - | 6 | - |
| Victim physical abuse^a^ | Yes | 7 | 11.9 | - | 6 | - |
| Victim psychological abuse^a^ | Yes | 6 | 10.2 | - | 6 | - |
| **Incident level** |  |  |  |  |  |  |
| Positive toxicology screen | Yes | 29 | 49.2 | <5 | 17 | 6 |
| Overkill (excessive force) | Yes | 6 | 10.2 | <5 | <5 | <5 |
| Location home | Yes | 42 | 72.4 | 7 | 31^b^ | <5^b^ |
| Mechanism blunt object | Yes | 12 | 20.3 | <5 | 10 | - |
| Mechanism bodily force | Yes | 18 | 30.5 | <5 | 12 | <5 |
| Mechanism sharp object | Yes | 21 | 35.6 | 6 | 12 | <5 |
| Single or multiple deaths | Multiple | 9 | 15.3 | <5 | 8 | - |
|  | Single | 50 | 84.7 | 10 | 29 | 8 |
| **Offender (n=57)** | **Categories** | **All offenders (n=57)** | **(%)** | **Acquaintance (n=11)** | **Intimate or familial (n=37)** | **Stranger (n=8)** |
| **Individual level** |  |  |  |  |  |  |
| Age group (10 year) | 15-24 | 8 | 14 | <5 | <5 | <5 |
|  | 25-34 | 13 | 22.8 | <5 | 10 | <5 |
|  | 35-44 | 9 | 15.8 | <5 | 7 | <5 |
|  | 45-54 | 14 | 24.6 | <5 | 9 | <5 |
|  | 55-64 | <5 | - | <5 | <5 | - |
|  | ≥65 | 6 | 10.6 | <5 | <5 | - |
| Mental illness, diagnosed | Yes | 36 | 63.2 | 7 | 22 | 7 |
| Historical violence exposure | Yes | 35 | 61.4 | 9 | 21 | 5 |
| History offending | Yes | 27 | 47.4 | 7 | 14 | 6 |
| History of substance use | Yes | 36 | 63.2 | 7 | 21 | 8^b^ |
| Suicidal ideation | Yes | 12 | 21.1 | <5 | 7 | <5 |
| Previous suicide attempt | Yes | 11 | 19.3 | <5 | 7 | <5 |
| Psychiatric treatment (not proximal) | Yes | 31 | 54.4 | 6 | 18 | 7 |
| Psychiatric treatment (proximal) | Yes | 24 | 42.1 | <5 | 15 | 6 |
| Service contact (any) | Yes | 43 | 75.4 | 8 | 27 | 7 |
| Service contact (proximal) | Yes | 26 | 45.6 | 6 | 14 | 5 |
| Sex | Female | 16 | 28.1 | <5 | 12 | <5 |
|  | Male | 41 | 71.9 | 10 | 25 | 5 |
| Mental illness, suspected | Yes | 22 | 38.6 | <5 | 14 | 5 |
| **Interpersonal level** |  |  |  |  |  |  |
| FV perpetrator^a^ | Yes | 11 | 19.3 | - | 10 | - |
| FV victim^a^ | Yes | 7 | 12.3 | - | 6 | - |
| Perpetrator controlling behaviours^a^ | Yes | 5 | 8.8 | - | 5 | - |
| Perpetrator assaulted victim^a^ | Yes | 6 | 10.5 | - | 5 | - |
| Perpetrator assaulted other family^a^ | Yes | 7 | 12.3 | - | 7 | - |
| Perpetrator threatened to harm/kill other family^a^ | Yes | 6 | 10.5 | - | 6 | - |
| Perpetrator verbally abused victim^a^ | Yes | 6 | 10.5 | - | 5 | - |
| Victim physical abuse^a^ | Yes | 5 | 8.8 | - | <5 | - |
| **Incident level** |  |  |  |  |  |  |
| Offender charged | Yes | 49 | 86 | 9 | 32 | 8 |
| Presence of alcohol or drugs | Yes | 24 | 42.1 | <5 | 16 | <5 |

a - only recorded for family violence-related homicides

b - p=<.05
